# Implications of Splicing Alterations in the Onset and Phenotypic Variability of a Family with Subclinical Manifestation of Peutz–Jeghers Syndrome: Bioinformatic and Molecular Evidence

**DOI:** 10.3390/ijms21218201

**Published:** 2020-11-02

**Authors:** Andrea Cerasuolo, Francesca Cammarota, Francesca Duraturo, Annamaria Staiano, Massimo Martinelli, Erasmo Miele, Paola Izzo, Marina De Rosa

**Affiliations:** 1 Molecular Biology and Viral Oncology Unit, Istituto Nazionale Tumori IRCCS “Fondazione G. Pascale”, 80131 Naples, Italy; andrea.cerasuolo91@gmail.com; 2 Department of Molecular Medicine and Medical Biotechnology, University of Naples Federico II, 80131 Naples, Italy; francesca.cammarota88@gmail.com (F.C.); francesca.duraturo@unina.it (F.D.); paola.izzo@unina.it (P.I.); 3 Ceinge Biotecnologie Avanzate, 80131 Naples, Italy; 4 Department of Translational Medical Sciences, Section of Pediatrics, University of Naples Federico II, 80131 Naples, Italy; staiano@unina.it (A.S.); massimo.martinelli@unina.it (M.M.); erasmo.miele@unina.it (E.M.)

**Keywords:** Peutz–Jeghers Syndrome (PJS), café au lait spots, splicing variants, Enhancer Splicing Element, presymptomatic diagnosis, cancer prevention, risk management

## Abstract

Peutz–Jeghers Syndrome (PJS) is an autosomal dominant pre-cancerous disorder caused in 80–90% of cases by germline mutations in the tumor suppressor gene *STK11*. We performed a genetic test of the *STK11* gene in two Italian young sisters suspected of PJS, since they showed pathognomonic café au lait spots in absence of other symptoms and familiarity. Sequencing of all exons of *STK11* gene and other 8 genes, suggested to be involved in hamartomatous syndromes, (*PTEN*, *BMPR1A*, *SDHB*, *SDHD*, *SMAD4*, *AKT1*, *ENG*, *PIK3CA*) led to the identification in both the probands of a novel germline silent mutation named c.597 G>A, hitting the last nucleotide of exon 4. Interestingly, genetic testing of the two probands’ parents showed that their unaffected father was carrier of this mutation. Moreover, he carried a second intronic substitution named c.465-51 T>C (rs2075606) which was not inherited by his daughters. We also observed that all the family members carrying the c.597 G>A mutation presented an aberrant splice variant of STK11 mRNA lacking exon 4. Furthermore, in silico analysis of c.465-51 T>C substitution showed that it may activate an Enhancer Splicing Element. Finally, qRT-PCR analysis of STK11 expression levels showed a slight downregulation of the wild type allele in the father and a 2-fold downregulation in the probands compared to the unaffected mother. Our results have led the hypothesis that the c.465-51 T>C intronic variant, which segregates with the wild type allele, could increase the splicing effectiveness of STK11 wild-type allele and compensate the side effect of the c.597 G>A splicing mutation, being responsible for the phenotypic variability observed within this family. This finding highlight the importance of RNA analysis in genetic testing, remarking that silent DNA variant can often be splicing variant involved in disease onset and progression. The identification of these variants has a crucial role to ensure an appropriate follow-up and cancer prevention in at-risk individuals.

## 1. Introduction

Peutz–Jeghers Syndrome (PJS) is a rare autosomal dominant inherited genetic disorder belonging to the family of hamartomatous polyposis syndromes, with an incidence rate ranging from 1:25,000 to 1:280,000 [1]. It is characterized by the development of noncancerous hamartomatous polyps in the gastrointestinal tract that may cause abdominal pain, self-limiting intussusception with bowel obstruction, and severe gastrointestinal bleeding during childhood [2,3]. PJS patients also present typical café au lait mucocutaneous spots around the eyes, mouth, buccal mucosa, nostrils, perineum, and fingers, and show an increased risk of developing different tumor types, including colorectal, pancreatic, ovarian, testicular, lung and cervical cancers, particularly at young ages [4,5,6].

Germline loss-of-function mutations affecting the serine-threonine kinase 11 (*STK11*) gene (also called *LKB1*), located on chromosome 19p13.3, have been shown to cause PJS in 50% to 90% of cases; however, about 25% of PJS patients present de novo mutations [7,8]. *STK11* is a tumor suppressor gene composed of nine exons encoding a kinase, which activates the AMP-activated protein kinase (AMPK) pathway, and is involved in cell cycle regulation, cell polarity, metabolism, and apoptosis [9]. Several heterozygous mutations in the *STK11* gene, including frameshift and missense mutations, duplications, deletions, and splicing errors causing PJS, have been identified [10,11,12,13,14,15]; however, a few studies evaluated the genotype-phenotype correlation in PJS, producing discordant results [16,17,18,19].

Interestingly, a great inter- and intrafamilial clinical variability has been described for PJS affected subjects, with some showing only mucocutaneous hyperpigmentation and others developing both pigmented lesions as well as gastrointestinal and/or extraintestinal symptoms [20]. Moreover, pigmental lesions may be absent or may be present in childhood but fade during adulthood [20]. However the mechanisms responsible for such phenotypic variability are still not clear.

Early PJS molecular diagnosis through *STK11* genetic testing is considered the most helpful strategy for effective PJS control and treatment, especially in families with no previous cases of the disease [2,21], allowing follow-up determination and cancer prevention in affected patients and in at risk family members.

In this study, we report the case of two young Italian sisters suspected of PJS with no family history of the disease, and perform *STK11* genetic screening test, investigating the molecular basis of genotype-phenotype correlation in this family.

## 2. Materials and Methods

### 2.1. Patients

**Figure 1:**
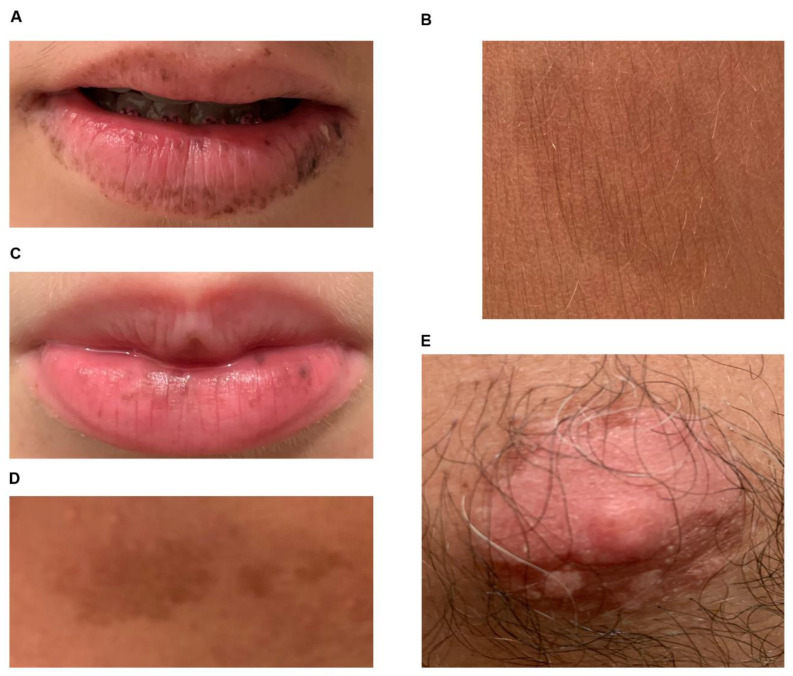
Café au lait spots of probands. (**A**) mucosal scattered black spots and typical café au lait spots on the lips of proband II-1; (**B**) café au lait cutaneous macules on the proband II-1 leg; (**C**) mucosal scattered black spots and typical café au lait spots on the lips of proband II-2; (**D**) café au lait cutaneous macules on the proband II-2 arm; (**E**) café au lait macules of the nipple areola of the probands’ father (subject I-1).

**Figure 2:**
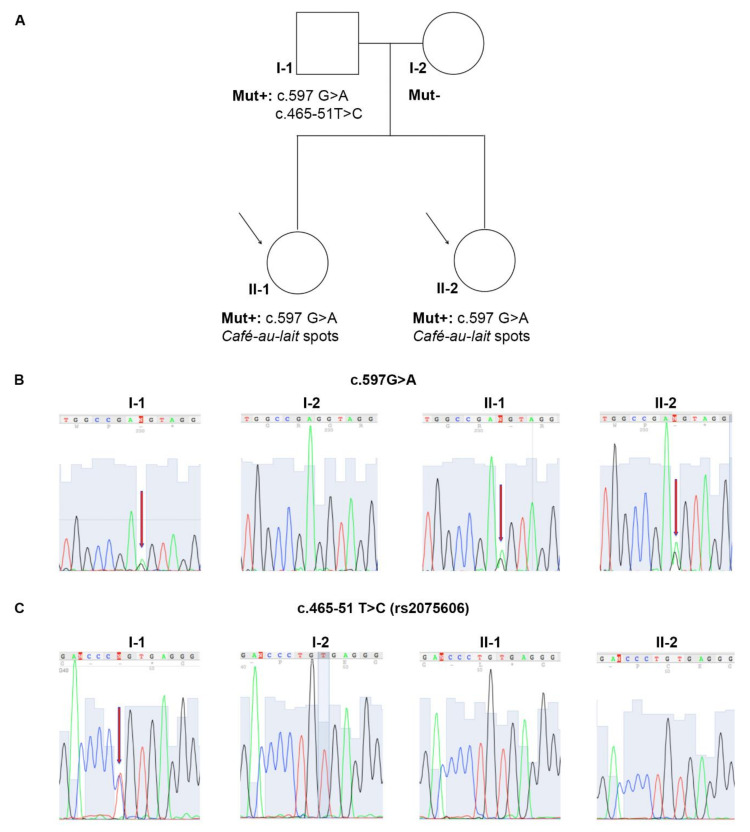
Molecular analysis of STK11/LKB1 gene. (**A**) Pedigree of the Peutz–Jeghers Syndrome (PJS) suspected family. (**B**) Electropherograms of STK11 sequence analysis showing the c.597G→A substitution in the exon 4. (**C**) Electropherograms of STK11 sequence analysis showing the c.465-51 T→C single-nucleotide polymorphism (SNP) in the intron 4. Red arrows indicate nucleotide changes; black arrows indicate the two PJS probands.

The patients were two 6- and 11-year-old sisters suspected of PJS. The clinical suspect of PJS was based on the presence of mucosal scattered black spots on their lips, and typical café au lait spots (Figure 1), in absence of intestinal polyps and other symptoms. No family history of PJS was described in the other members of the family except for the presence of café au lait macules on the nipple areola of the proband father (subject I-1) (Figure 1E and Figure 2A).

The patients’ parents received clinical and genetic counseling and provided informed consent to molecular screening.

### 2.2. Genetic Analysis

Genomic DNA was extracted from peripheral blood lymphocytes of the probands and their parents as previously described [22]. Briefly, 2 mL of the patients’ blood were resuspended in five volumes of red blood lysis buffer (0.15M NH_4_Cl_2_ and 0.17M Tris-HCl, pH 7.65), incubated at 37° for 15 min, and centrifuged. After centrifugation, the lymphocyte pellets was resuspended in a half volume of DNA extraction buffer (1M Tris HCl, 0.5M EDTA, and 5M NaCl) and digested with Proteinase K and 10% SDS at 60 °C for 10 min. Next 6M NaCl was added, then the samples were centrifuged and the DNA in the supernatants was precipitated with two volumes of absolute ethanol. Finally, isolated DNA was resuspended in an appropriate volume of deionized sterile water. DNA quality and concentration were spectrophotometrically assessed using the Nanodrop2000 (Thermo Fisher Scientific, Waltham, MA, USA).

Exons 1 to 9 of STK11 were amplified by PCR in a 50 μL reaction mixture containing 100 ng of genomic DNA, 0.4 μL of Taq DNA polymerase, 5 μL 10X buffer (200 mM Tris-HCl pH 8.4, 500 mM KCl), 50 mM MgCl2, 1 μL 1 mM dNTPs, and 1 μL of each specific forward and reverse primers (Table 1).

The amplification protocol was as follows: 4 min at 95 °C, then 35 cycles of 30 s at 95 °C, 30 s at 60 °C, and 45 s at 72 °C, and a final extension of 7 min at 72 °C. All the reactions were performed in the thermal cycler MyCycler (Bio-Rad, Hercules, CA, USA). Amplified fragments were run on 1x agarose gel and visualized with ethidium bromide, then purified with QIAquick PCR Purification Kit (Qiagen, Hilden, Germany) according to the manufacturer’s instructions. After purification, amplicons were subjected to Sanger sequencing. The analysis of the STK11 sequences was performed by alignment with those present in the GenBank database using the BLASTn software (http://www.ncbi.nlm.nih.gov/blast/html). The accession number of the reference sequence used was NC_000455.4.

The genetic analysis of STK11 was performed, also, in the probands’ parents following the same procedure.

Furthermore, a panel of ten genes, including *STK11/LKB1*, *PTEN*, *BMPR1A*, *SDHB*, *SDHD*, *SMAD4*, *AKT1*, *ENG*, and *PIK3CA* was analyzed in the probands and their father, by next generation sequencing (NGS), using AmpliSeq Library PLUS for Illumina (catalog ID: 20019101), according to the manufacturers’ instructions. The pooled and barcoded libraries were subsequently sequenced using MiSeq sequencer (Illumina Inc.). Variant calling and analysis were performed on Base Space Sequence HUB/variant interpreter Software (basespace.illumina.com).

### 2.3. STK11 mRNA Qualitative and Quantitative Analysis

Total RNA was extracted from peripheral blood lymphocytes using the QIAzol Lysis Reagent (Qiagen) according to the manufacturer’s instructions and quality and concentration of extracted RNA was determined using the Nanodrop2000. After treatment with DNase I (RNase free) (New England BioLabs, Ipswich, MA, USA), 1 µg of total RNA was reverse transcribed using the SuperScript III Reverse Transcriptase Kit (Invitrogen, Waltham, MA, USA) in a 20 µL reaction volume containing 500 ng random primers, as previously described [23].

The cDNA samples were amplified for STK11 by RT-PCR in a 50 μL reaction mixture, including 1 μL of cDNA, 0.4 μL of Taq DNA polymerase, 5 μL of 10X buffer, 50 mM MgCl2, 1 μL of 1 mM dNTPs and 1 μL of each forward and reverse primers. Specifically, primers 2c-FP (5′-GGATGTGTTATACAACGAAG-3′) and 6cRP (5′-TTCTCAAACAACTTGTAGATG-3′) were used for the amplification reaction. The reactions protocol was as follows: 5 min at 94 °C, then 33 cycles of 30 s at 94 °C, 20 s at 60 °C, 45 s at 72 °C, and a final elongation of 3 min at 72 °C.

As for genomic DNA analysis, amplicons were resolved and visualized on 1x agarose gel, purified with the QIAquick PCR Purification Kit and subjected to Sanger sequencing for nucleotide sequence analysis by BLASTn online software.

Furthermore, cDNA was amplified for STK11 by Real Time-PCR, as previously described [24], using the iCycler iQ Real-Time Detection System (Bio-Rad) in reaction mixture containing 0.5 μL cDNA, 7.5 μL SYBR Green PCR Master Mix (Bio-Rad), 1 μL of 2-cFP forward primer (5′-GGATGTGTTATACAACGAAG-3′) and of 4cRP reverse primer (5′-AGGTCGGAGATTTTGAGGGT-3′) and nuclease-free water in a final volume of 15 μL. The expression of *STK11* was analyzed with the 2^ΔΔCt^ method using the β-glucuronidase (GUS) housekeeping gene as internal calibrator.

### 2.4. In Silico Sequence Analysis

The presence of splicing regulating sequences within the exon/intron 4 region of the *STK11* gene was investigated in all DNA samples using the “ESEfinder” (http://exon.cshl.edu/ESE/) and “Human Splicing Finder” (http://www.umd.be/HSF/) online software [25,26,27], as previously described [28]. Default threshold values were used to evaluate nucleotide variants in exonic and intronic splicing motifs.

### 2.5. Statistical Analysis

Real time data were obtained from at least three independent experiments and are reported as the mean ± SEM. Statistical differences between groups were determined by the repeated measures multiple comparisons, Kruskal–Wallis test, one-way ANOVA, at a significance level of *p* < 0.05.

## 3. Results

### 3.1. DNA Analysis

The genetic analysis of *STK11*, performed in both the probands revealed the presence of a germline heterozygous G to A transition named c.597G>A, affecting the last nucleotide of exon 4 (Figure 2B). The genetic test in the probands’ parents showed that the mutation was present also in their father, but was absent in their mother (Figure 2B). Interestingly, a second heterozygous T to C variant lying in *STK11* intron 4, named c.465-51 T>C (rs2075606), was also identified in the father; however, it was neither detected in the mother nor in the probands (Figure 2C). No other pathogenic variants or variants of unknown significance were found by NGS analysis in the other genes investigated.

### 3.2. STK11 Isoforms Characterization and Expression Analysis

**Figure 3:**
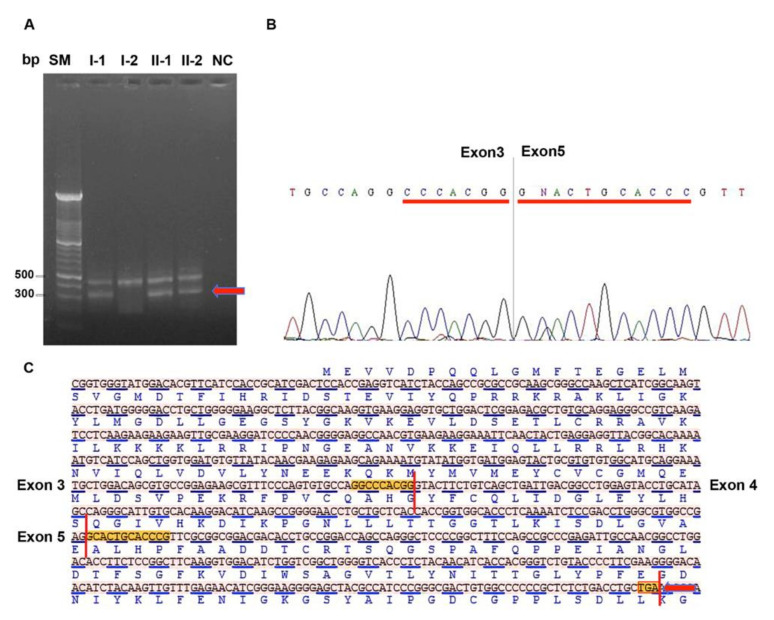
Identification of the altered splicing isoform. (**A**) RT-PCR analysis of the cDNA region encompassing exons from 2 to 6. The arrow indicates the abnormal mRNA fragment showing lower molecular weight. (**B**) Electropherogram showing a *STK11* isoform lacking exon 4 and the formation of a new junction between exons 3 and 5. (**C**) STK11 cDNA sequence in FASTA format showing junctions between exons 3–4 and 4–5; skipping of exon 4 generate a reading frame-shift and formation of a premature stop codon highlighted in the red box and indicated with red arrow. Bp: base pair, SM: size marker, I-1, I-2, II-1 and II-2: subjects of PJS family as reported in pedigree of Figure 2A, NC: RT-PCR negative control without template.

The amplification of STK11 messenger fragment encompassing coding region from exon 2 to exon 6, showed the presence of two STK11 isoforms with different molecular weight in the probands and their father (Figure 3A).

Direct sequencing of these two isoforms revealed that the isoform with higher molecular weight was the wild-type form, while the isoform with lower molecular weight lacked the exon 4 with consequent generation of a premature stop codon (Figure 3B,C).

**Figure 4:**
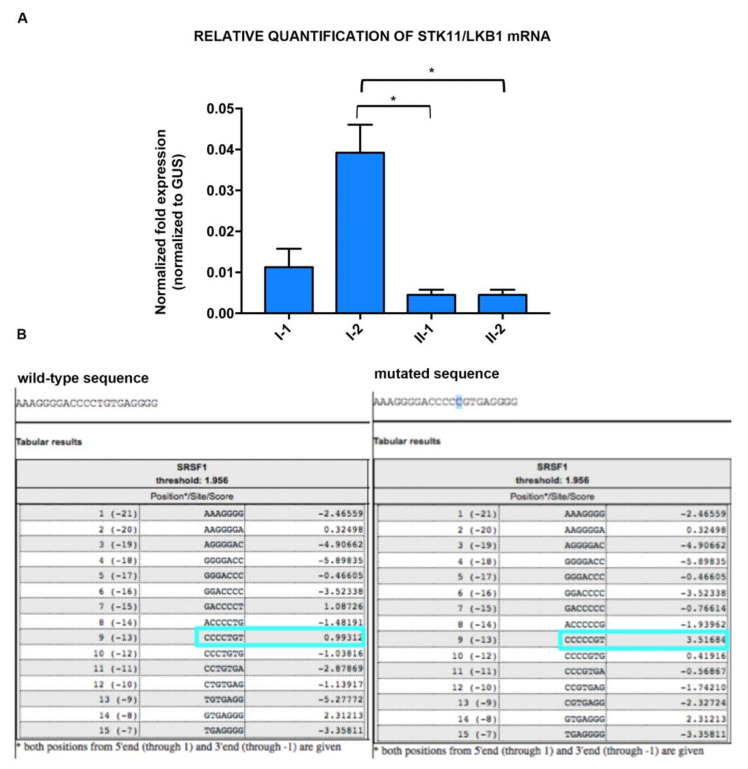
Potential role of the c.465-51 T→C SNP. (**A**) Real-Time PCR of *STK11* mRNA; Bar graphs represent mean ± SEM (three independent determinations) of normalized STK11 expression to glucuronidase mRNA (Dct); *: significance level of *p* < 0.05 vs. healthy subject I-2; repeated measures multiple comparisons Kruskal–Wallis test one-way ANOVA has been used for the analysis. I-1, I-2, II-1 and II-2: subjects of PJS family as reported in pedigree of Figure 2A. (**B**) ESEfinder in silico analysis of *STK11* c.465-51T→C variant in intron 4 showing activation of a binding site for the serine/arginine-rich splicing factor (SRSF1) splicing factor. The light blue boxes indicate SRSF1 protein binding score calculated for wild type and mutant sequences.

The analysis of *STK11* expression by Real Time PCR showed a 2-fold downregulation of the wild-type *STK11* isoform in the probands compared to the unaffected mother, but only a slight downregulation in the father (Figure 4A). These observations were supported by statistical analysis of STK11 mRNA real time quantification data that showed significance for subjects II-1 and II-2 vs. subject I-2, while no significance was observed nether for subject I-1 vs. subject I-2, nor for subjects II-1and II-2 vs. subject I-1.

### 3.3. In Silico Analysis of STK11 Splicing Sequence

The in silico analysis of *STK11* exon/intron four boundaries by Human Splicing Finder showed that the c.597G>A substitution disrupted the GT splice donor site located at the 5′-end of *STK11* intron 4, affecting the splicing of STK11 mRNA (data not shown).

Moreover, the ESEfinder analysis of *STK11* intron 4 sequence identified an exonic splicing enhancer (ESE) motif at nucleotide–13(CCCCTGT) recognized by the serine/arginine-rich splicing factor (SRSF) 1 and showed that the c.465-51 T>C SNP (rs2075606), within the ESE, increased the SRSF1 binding affinity to this sequence (Figure 4B).

## 4. Discussion

The early clinical diagnosis of PJS may be difficult due to its variable penetrance and the lack of knowledge of the mechanisms underlying such variability [29]. For this reason, *STK11* genetic screening test is recommended for all subjects suspected of PJS, also in absence of PJS family history [29,30].

In this study, we analyzed *STK11* sequence in two young sisters suspected of PJS showing mucocutaneous hyperpigmentation typical of PJS and identified a novel germline heterozygous G to A transition (c.597G>A) inactivating the splice donor site at the 5′-end of *STK11* intron 4.

Mutations of nucleotides located at the exon–intron junctions generally affect the splicing process, which is necessary for the removal of introns from pre-mRNAs and the production of mature mRNAs, causing the production of anomalous mRNA isoforms involved in carcinogenesis, as well as other genetic disorders [31,32]. Most of splicing reactions are mediated by the major spliceosome complex, composed of five small nuclear ribonucleoproteins (U1, U2, U4, U5, and U6) and more than 100 peptides that bind the splice donor and acceptor sites (located at the 5′- and 3′-end of introns, respectively) as well as the branch point (located within introns) [33]. Moreover, different combinations of splice donor and acceptor sites can be recognized by splicing factors, leading to the production of different transcripts isoforms from a single mRNA molecule [34]. Regulatory splicing factors classified as heterogeneous ribonucleoproteins (hnRNPs) and SRSFs modulate the splicing efficiency by recognizing exonic splicing enhancers (ESEs) and intronic splicing enhancers (ISEs) or exonic splicing silencers (ESSs) and intronic splicing silencers (ISSs), respectively [35]. Exonic splicing enhancer has also abundant in introns with weak donor or acceptor sites [36].

The c.597G>A substitution detected in both two probands results in the production of an abnormal STK11 mRNA isoform which lacks exon 4, generating a premature stop codon and potentially leading to the translation of a truncated STK11 protein. This variant was also detected in the probands’ father, who, in our knowledge, did not report any PJS symptoms or stigma. Notably, he presented a heterozygous T to C variant [c.465-51 T>C] in *STK11* intron 4, which was absent in the probands. The ESEfinder analysis of *STK11* gene sequence showed that the c.465-51 T>C variant increases the binding affinity of the SRSF1 splicing factor for the ESE motif located in intron 4. Through this mechanism the rs2075606 polymorphism may be responsible for higher *STK11* mRNA expression in the probands’ father compared to his daughters, attenuating the side effect of the pathogenetic c.597G>A variant and contributing to the PJS phenotypic variability in the family. This is only a speculative consideration because we couldn’t in vivo demonstrate that expression of the allele with c.465-51 T>C is higher than that of other allele and further experiments are needed to shed light on this observation. Furthermore, in our opinion, the rs2075606 polymorphism could represent an attenuator factor peculiar for the specific pathogenic variant observed in this family, the c.597G>A, because it causes exon 4 skipping and for similar pathogenic variant with the same effect on mRNA processing

Few studies investigated the role of splicing mutations and aberrant *STK11* transcript isoforms in the pathogenesis of PJS [10,11,37,38,39,40,41]. Yu-Liang et al. identified a higher percentage of STK11 splicing mutation in a Chinese patient cohort; however, no correlation was found between splicing errors and clinical manifestations, including cancer type occurrence [13]. The contribution of STK11 splicing mutations and isoforms in PJS pathogenesis and phenotypic variability needs further investigation.

In conclusion, the present study highlighted the importance of early genetic testing of *STK11* gene in young PJS patients, especially in absence of PJS family history, and provided further evidence on the role of splicing modulation in the onset and phenotypic variability of PJS.

## Figures and Tables

**Table 1:** Oligonucleotide sequence of primer pairs used for *STK11* genetic analysis.

Primers	Sequences (5′–3′)
1FP	5′-AACACAAGGAAGGACCGCTAC-3′
1RP	5′-GACAGAACCATCAGCACCGTGAC-3′
2FP	5′-CCTCCAGAGCCCCTTTTCT-3′
2RP	5′-AAGGAGACGGGAAGAGGAC-3′
3aFP	5′-CCTCCAGAGCCCCTTTTCT-3′
3aRP	5′-ATCAGGACACAAGCAGTGTGGC-3′
3bFP	5′-CCCCCTGAGCTGTGTGTC-3′
3bRP	5′-AGTGTGGCCTCACGGAAA-3′
4FP	5′-GTGTGCCTGGACTTCTGTGA-3′
5RP	5′-GAGTGTGCGTGTGGTGAGTG-3′
6FP	5′-AACCACCTTGACTGACCACGC-3′
6RP	5′-GACACACCCCAACCCTACATTTCTG-3′
7FP	5′-CGCCCCAGGGGGAATCCTC-3′
7RP	5′-CTAGCGCCCGCTCAACCAG-3′
8FP	5′-GGAGCTGGGTCGGAAAACTGGA-3′
8RP	5′-TGCTCCCGTGGGACATCCTG-3′
9aFP	5′-GTAAGTGCGTCCCCGTGGTG-3′
9aRP	5′-CGGTCACCATGACTGACTAGC-3′
9bFP	5′-CCTGTGGCTCTGGGGTTGC-3′
9bRP	5′-CACGGCTGGCTGTGGCATC-3′

## Data Availability

All data generated or analyzed during this study are included in this article.
